# Alcohol Intake Among Breast Cancer Survivors: Change in Alcohol Use During a Weight Management Intervention

**DOI:** 10.2196/cancer.6295

**Published:** 2016-11-09

**Authors:** Tera L Fazzino, Kimberly Fleming, Christie Befort

**Affiliations:** ^1^ Department of Preventive Medicine and Public Health University of Kansas Medical Center Kansas City, KS United States; ^2^ Department of Psychiatry and Behavioral Sciences University of Kansas Medical Center Kansas City, KS United States

**Keywords:** alcohol drinking, breast cancer, weight loss, weight reduction programs, obesity

## Abstract

**Background:**

Daily alcohol intake in quantities as small as half a drink/day significantly increases the risk of breast cancer recurrence for postmenopausal survivors. Interventions designed to modify alcohol use among survivors have not been studied; however, lifestyle interventions that target change in dietary intake may affect alcohol intake.

**Objective:**

To evaluate change in alcohol use during a weight loss intervention for obese, rural-dwelling breast cancer survivors.

**Methods:**

Data were derived from an 18-month trial that included a 6-month weight loss intervention delivered via group conference calls, followed by a 12-month randomized weight loss maintenance phase in which participants received continued group calls or mailed newsletters. Participants who reported regular alcohol use at baseline (N=37) were included in this study.

**Results:**

Mean daily alcohol intake significantly decreased from baseline to 6 months during the weight loss intervention (19.6-2.3 g; *P*=.001). Mean alcohol intake did not significantly increase (*b*=0.99, *P*=.12) during the weight loss maintenance phase (months 6-18) and did not depend on randomization group (*b*=0.32, *P*=.799).

**Conclusions:**

Findings provide preliminary evidence that a weight loss intervention may address obesity and alcohol use risk factors for cancer recurrence. Minimal mail-based contact post weight loss can maintain alcohol use reductions through 18 months, suggesting durability in these effects. These results highlight a possibility that lifestyle interventions for survivors may modify health behaviors that are not the main foci of an intervention but that coincide with intervention goals.

**Trial Registration:**

Clinicaltrials.gov NCT01441011; https://clinicaltrials.gov/ct2/show/NCT01441011 (Archived by WebCite at http://www.webcitation.org/6lsJ9dMa9)

## Introduction

Results of comprehensive meta-analyses have indicated that daily alcohol use, even in small quantities, is associated with an increased risk of both developing breast cancer and breast cancer recurrence [1-3]. There is a significant dose-response relationship between daily alcohol use and breast cancer risk [4], and for every 1 drink per day (10 g) increase in alcohol consumption, there is a 12% increased risk of breast cancer [1]. As little as 5-6 g of daily alcohol intake has been found to significantly increase the risk of developing breast cancer [5] and breast cancer recurrence [3,6]. Mechanisms responsible for the increased risk may be due to increased estrogen and androgen levels [7,8] or increased levels of plasma insulin-like growth factors produced by the liver following alcohol consumption [9]. In addition, alcohol-related breast cancer risk may be compounded for individuals who have other lifestyle risk factors, such as obesity [6]. Both the American Cancer Society [10] and the National Institute on Alcohol Abuse and Alcoholism (NIAAA) [11] recommend that women consume no more than 1 drink per day, and the recently published European Code against Cancer 4th Edition stresses that zero alcohol intake is recommended for cancer prevention [12]. Newly proposed UK drinking guidelines specify that individuals should have some non-drinking days each week [13].

Multiple breast cancer survivor cohort studies have found that alcohol use prevalence among female survivors is similar to that in the general US female population; 15-23% of survivors drink in excess of low-risk drinking guidelines [14-16]. Although cancer diagnosis may be a teachable moment to prompt health behavior change, 1 large-scale, population-based study among cancer survivors have found no significant long-term changes in daily alcohol consumption pre- to post-cancer diagnosis [17]. One smaller breast cancer cohort study found that the prevalence of heavy episodic drinking decreased following the diagnosis, however levels of daily alcohol use were not reported [18].

Interventions designed to modify alcohol consumption among breast cancer survivors have not previously been studied; however, it is possible that lifestyle interventions that target weight loss and promote change in dietary intake may affect alcohol consumption. Alcohol has an energy density that is second only to fat [19] and reducing alcohol intake would coincide with lifestyle intervention goals of calorie restriction and energy balance. The purpose of this study was to examine change in alcohol use among obese, rural breast cancer survivors participating in an 18-month weight management intervention who reported regular alcohol use at baseline. Specifically, we evaluated initial change in alcohol use during a 6-month weight loss intervention phase and the durability of these effects during a 12-month weight loss maintenance phase.

## Methods

### Parent Study Overview

The parent study from which the data for this study were derived compared group phone-based counseling with mailed newsletters on weight loss maintenance following successful weight loss among rural, obese breast cancer survivors. The study has previously been detailed [20,21] and is briefly described here. Participants (N=210) were recruited in 8 cohorts from oncology centers in the Midwestern United States. Participants were female breast cancer survivors aged 75 years or younger, with a BMI between 27 and 45 kg/m^2^, who had been diagnosed with Stages 0-III disease within the past 10 years, were at least 3 months out of treatment at the time of enrollment, had physician clearance to participate, and resided in rural areas [22]. Current alcohol or drug abuse [23] was an exclusion criterion, however no participants were excluded for this reason [20]. The trial included a nonrandomized 6-month weight loss phase (0-6 months), where all participants received weekly group phone sessions, followed by a 12-month randomized weight loss maintenance phase (6-18 months) in which participants who lost ≥5% of their baseline weight were randomized to continued group phone sessions or a newsletter comparison condition. The Institution’s Human Subjects Committee approved the study.

### Intervention

During the weight loss phase, groups of 10-15 participants met for weekly counseling sessions via conference call with a group counselor (registered dietitian or psychologist). Intervention and technology accessibility were a primary focus with our rural-dwelling sample; thus, we used a conference call format that did not require participants to have regular access to a computer or mobile phone. Participants joined the conference sessions by calling into a toll free, 1-800 number accessible from any landline, mobile phone, or Internet-based telephone system (such as Skype or Google Voice). During the calls, participants were instructed to follow a structured meal plan that included whey-based protein shakes, prepackaged portion-controlled meals (eg, Lean Cuisines), and at least five fruits or vegetables per day. Snacks that were not fruits or vegetables, as well as eating out were discouraged. Participants were instructed to gradually increase their physical activity each week with the goal of completing 225 min/week of moderate-intensity physical activity by week 12.

The weight loss phase focused on behavioral skills for healthy eating, increasing physical activity, and self-monitoring daily calorie intake and physical activity. Two sessions focused on evidence-based nutrition recommendations for breast cancer survivors specifically, including alcohol use as a risk factor for breast cancer recurrence and American Cancer Society recommendations for consuming 1 or less alcoholic drinks per day. During this session, leaders also discussed the calorific content of alcoholic beverages as related to participants’ goals of limiting their total calorific intake and losing weight.

During the weight loss maintenance phase, participants randomized to the group phone condition continued to meet every other week via conference calls, while participants in the newsletter control condition received bi-weekly newsletters highlighting the same content. The maintenance phase focused on problem solving related to maintaining diet and physical activity behaviors and increasing motivation, and did not specifically address alcohol use.

### Data and Measures

Multiple pass 24-h diet recalls were collected from participants at baseline, 6 months, 12 months, and 18 months. United States Department of Agriculture (USDA) multiple pass 24-h dietary recalls are the gold standard for measuring typical dietary intake [24] and are also valid as a measure of alcohol consumption when compared with a 1 week recall of alcohol use [25].

Of the 210 participants enrolled in the parent study, 42 participants (20%) reported daily alcohol intake of at least 5 g at baseline. Of the 20% (42/210), 11% (5/42) of the participants drank more than 1 standard drink per day and 9% drank about half a standard drink per day.

Thirty-seven participants provided 2 valid diet recalls at both baseline and 6 months that reflected their typical dietary intake and thus were included in these analyses. A total of 277 diet recalls were collected from the sample out of a possible 296, representing a 94% assessment compliance rate. Sixty-seven recalls were completed at 12 months and 62 at 18 months.

### Statistical Analyses

A paired-samples *t* test was utilized to test whether daily alcohol grams significantly decreased during the weight loss phase (0-6 months). Generalized estimating equation (GEE) models were used to examine change in daily grams of alcohol intake during the randomized weight loss maintenance phase (6, 12, and 18 months), while accounting for the within-subject report correlation structure of the longitudinal data [26]. GEE Model 1 controlled for age, college education, and randomization group. A second GEE model included an interaction between time and randomization group to evaluate whether change in alcohol use depended on randomization group. For both GEE models, we determined that an exchangeable correlation structure fit the data best based on the Quasi-Likelihood under the Independence Model Criterion (QIC) [27]. All statistical analyses were conducted using SPSS version 22 (IBM Corporation) [28].

## Results

Demographic characteristics of participants (N=37) are presented in Table 1. The majority of participants (28/37; 75%) were obese and 25% (9/37) were overweight. Participants were a mean of 3.5 years beyond cancer treatment at the start of the study. At 6 months, participants lost a mean of 12.9 kg (SD 5.4), corresponding to a mean of 14.4% (SD 5.9) of their baseline weight. By 18 months, participants regained a mean of 3.3 kg (SD 5.2) of the weight they lost.

**Table 1:** Participant Demographics (N=37).

Variable	Mean (SD) or %
Age	57.8 (7.9)
Marital status (% Married or cohabitating)	89
Race or Ethnicity (% white)	100
Education (% BA degree or higher)	31
Employed full-time	75
**Weight variables**	
Body mass index	33.3 (4.3)
Overweight	75
Obese	25
**Cancer variables**	
Age at diagnosis	54.0 (8.3)
Time since cancer treatment (years)	3.5 (2.4)
**Stage**	
0	19
I	41
II	27
III	13
**Treatment received**	
Breast-conserving surgery	57
Mastectomy	43
Radiation	71
Chemotherapy	57
Anti-hormone Therapy	82
**Alcohol variables**	
Grams of alcohol per day	19.6 (17.85)
10 g of alcohol or more per day^a^	62
14 g of alcohol or more per day^b^	51
Daily heavy alcohol use (>3 drinks/42 g)^c^	15

^a^10 g=definition of 1 standard drink per meta-analyses on alcohol use and breast cancer risk.

^b^14 g=definition of 1 standard drink per the US National Institute on Alcohol Use and Alcoholism (NIAAA).

^c^Daily heavy alcohol use = alcohol consumption in excess of NIAAA guidelines for low-risk drinking for women.

**Table 2:** Change in alcohol use during a weight management intervention.

Alcohol intake	Full sample (N=37)		Group phone counseling (n=17)	Newsletter comparison (n=20)	*P* value (between groups)
	Mean (SD)	*P* value (full sample)	Mean (SD)	Mean (SD)	
Baseline daily alcohol use (grams)	19.61 (17.8)	<.001	17.0 (13.5)	20.5 (20.9)	.35^c^
Daily alcohol use at 6 months (grams)	2.28 (5.1)	.001^a^	1.3 (2.4)	2.7 (6.1)	.25^d^
Daily alcohol use at 18 months (grams)	4.20 (9.2)	.12^b^	3.52 (7.9)	5.1 (10.7)	.80^e^

^a^Paired samples *t* test of change in alcohol use from baseline to 6 months during the weight loss intervention phase.

^b^Generalized estimating equation (GEE) model of change in alcohol use during weight loss maintenance (months 6-18); full results of this model are presented in Table 3.

^c^Independent samples *t* test of baseline alcohol use by group.

^d^Repeated measures analysis of variance testing change in alcohol use by group during weight loss phase (baseline to 6 months)

^e^GEE model of change in alcohol use during weight loss maintenance phase, testing for moderating effects of randomization group.

**Table 3:** Change in alcohol use during a weight loss maintenance intervention using a generalized estimating equation model.

Variable	DV^a^: Daily alcohol intake (in grams)			
	B^b^	SE^c^	*P* value	95% CI
Time	0.99	0.64	.119	−0.26 to 2.26
Age	0.14	0.13	.268	−0.11 to 0.40
Education (college degree/not)^d^	2.27	2.66	.393	−2.94 to 7.46
Randomization group (intervention/control)^e^	−1.70	1.91	.373	−5.45 to 2.05

^a^DV: dependent variable.

^b^B: unstandardized regression coefficient.

^c^SE: standard error.

^d^No 4-year college degree was the reference category.

^e^The mail-based control group was the reference category.

Participants drank a mean of 19.6 g of alcohol per day at baseline (SD 17.85; Range 5.5-92.3), which corresponds to 1.4 US standard drinks [11]. Participants consumed a mean of 6.4% (SD 5.9; Range 1.4-32.8%) of their daily calories from alcohol at baseline.

Change in alcohol use during the study is presented in Table 2. Mean daily alcohol grams significantly decreased from baseline to 6 months during the weight loss intervention (19.6-2.3 g; *t*_36_=6.07, *P*=.001, 95% CI 11.5-23.1), corresponding to a mean decrease of 1.2 US standard drinks.

During the weight loss maintenance phase, alcohol intake did not significantly increase when controlling for randomization group, age, and education level (Table 3). For every unit increase in time, participants consumed 0.99 more grams of alcohol. Change in alcohol use during Phase II did not depend on randomization group when accounting for potential confounding variables (*b*=.32, SE=1.27, *P*=.799, CI −2.16 to 2.81).

Results indicate that the significant and clinically meaningful decrease in alcohol intake that occurred during the weight loss phase was maintained during the weight loss maintenance phase among participants in both the group phone counseling condition and the newsletter condition.

## Discussion

### Principal Findings

The risk for breast cancer recurrence increases significantly for postmenopausal breast cancer survivors who regularly drink alcohol, even at very low levels [3,6]. This study is the first to our knowledge to report on significant change in alcohol use among breast cancer survivors following a behavioral weight loss intervention. The findings suggest that among postmenopausal, obese survivors who drank regularly at baseline, participation in a behavioral weight control intervention was associated with significantly decreased alcohol intake in addition to clinically meaningful weight loss. Importantly, participants decreased their alcohol use to less than the 5 g per day level that is associated with increased recurrence risk [3,6]. Thus, findings suggest that a behavioral weight control intervention may simultaneously change multiple lifestyle risk factors for breast cancer recurrence.

A growing body of literature has focused on changing multiple health behaviors including dietary intake, physical activity, and substance use, either simultaneously or sequentially using dually focused interventions [29]. Our results are unique as they suggest that an intervention designed to address a single issue may influence change in other risk behaviors that coincide with the overall goals of the intervention. At a population level, epidemiologists have observed such tag-along behavior change effects in some circumstances in which individuals attempt to change a single behavior; for example, Brown et al found that individuals in England who reported attempted to quit smoking in a cross-sectional survey also reported fewer heavy drinking episodes during that time [30]. Our findings are novel in that we observed these effects longitudinally in an individually focused clinical intervention trial. In this regard, it is also important to emphasize that our intervention was focused on improving coping skills and self-efficacy, problem solving, addressing environmental barriers, and identifying triggers, all of which can also be helpful in decreasing alcohol use.

Our findings also indicate that alcohol use reductions were durable over an 18-month period. The decrease in alcohol use achieved during the weight loss intervention was maintained during the weight maintenance phase across both conditions, despite some weight regain. These sustained reductions in alcohol intake may in part be attributed to lower drinking levels at baseline, and also because decreasing alcohol intake was in accordance with the dietary goals of restricting overall calorific intake, and the intervention addressed alcohol consumption as an independent risk factor for recurrence.

The prevalence of daily drinking among the total sample from the parent study was in line with national estimates (20%) [14-16]; however, drinking quantity was lower than that found in other survivor samples. Specifically, 15-18% of breast cancer survivors from large cohort samples reported consuming 1+ drinks per day [15,16], compared with our sample where 11% of participants drank more than 1 standard drink per day and an additional 9% drank about half a drink per day. This difference may be due to our rural sample of overweight or obese women interested in losing weight. Rural survivors have been found to drink less than the survivors in urban environments [31], and our findings were similar with Weaver et al (2013) having estimated that 13% of rural survivors drink daily.

### Limitations

This study had several limitations. First, the sample size in our study was small. However, given that this study is the first to report a reduction in survivors’ alcohol use during a behavioral intervention, our findings provide valuable initial information on change in survivors’ health risk behaviors and suggest that future research on this topic using larger samples is warranted. In addition, all participants completed the weight loss intervention, thus we did not have a nonweight loss control condition with which to compare initial change in alcohol use. However, 1 large-scale study found that survivors did not change daily alcohol use patterns long-term [17], suggesting that daily alcohol use among survivors may be stable long-term without behavioral intervention. Second, the findings may not be generalizable to nonrural survivors or those with heavier drinking at baseline. Findings also may not be generalizable to more racially and ethnically diverse samples; a limitation of our study was that the sample comprised all Caucasian women. Finally, alcohol use was measured using 24-h dietary recalls, thus it was not possible to evaluate heavy episodic drinking rates.

Future research in this area should incorporate additional alcohol measures and investigate whether weight loss interventions decrease alcohol use among breast cancer survivors who are heavier drinkers. Researchers should examine the effects of heavy alcohol use on obesity to determine whether alcohol use as a cancer recurrence risk factor contributes to obesity, another risk factor. Relatedly, researchers could investigate whether heavy alcohol use hinders weight loss and contributes to weight regain. We were not able to address this question because participants in this study reduced their alcohol use to less than what would be expected to interfere with weight loss or maintenance.

### Implications

Our findings provide preliminary evidence that a weight loss intervention for obese breast cancer survivors may address both obesity and alcohol use risk factors for breast cancer recurrence with durability in alcohol reduction even with minimal content directly targeting alcohol use. Thus, lifestyle interventions for survivors may modify health behaviors that are not the main foci of intervention but that coincide with the overall goals of an intervention. This study highlights the possibility of improving the health of survivors using behavioral interventions focused on developing behavioral skills that might generalize to related risk behaviors.

## Abbreviations

GEE: Generalized estimating equation.
